# Post-hepatectomy tolvaptan-induced hypernatremia in a hepatocellular carcinoma patient with cirrhosis: a case report

**DOI:** 10.1186/s40792-020-00825-w

**Published:** 2020-03-30

**Authors:** Hiroya Iida, Hiromitsu Maehira, Haruki Mori, Tsuyoshi Maekawa, Masaji Tani

**Affiliations:** Department of Surgery, Shiga University of Medical Science, Seta Tsukiniwa-Cho, Otsu, Shiga 520-2192 Japan

**Keywords:** Hypernatremia, Tolvaptan, Hepatectomy, Hepatocellular carcinoma, Cirrhosis

## Abstract

**Background:**

Tolvaptan is used in Japan to reduce fluid retention caused by cirrhosis. However, hypernatremia is one of the most important side effects. This report is the first case report of a patient who developed hypernatremia after tolvaptan administration in the early stages following hepatectomy.

**Case presentation:**

A female patient in her 60s, who was admitted to the psychiatric department of a different hospital for bipolar disorder, developed hepatocellular carcinoma with cirrhosis. She was transferred to our hospital, and hepatectomy was performed in October 2019, after which pleural effusion and severe edema due to fluid retention were evident. Thus, the patient was started on tolvaptan (7.5 mg/day) from postoperative day (POD) 1. The patient began to experience disturbance of consciousness after POD 4. On the fifth day, the serum sodium (Na) level increased to 174 mEq/L, and hypernatremia was diagnosed. The Na level gradually improved with fluid infusion therapy, dropping to preoperative levels on the ninth day; her consciousness also gradually improved.

**Conclusions:**

Tolvaptan administration must be performed under strictly controlled conditions, followed by careful observation during the early postoperative period, when the patient’s physical status is unstable.

## Background

Tolvaptan is a drug that selectively controls water reabsorption in the kidney. It is an antagonist of the vasopressin-2 receptor (a protein expressed in the basolateral membrane of the principal cells of the collecting duct) and acts by inhibiting the aquaporin-2-mediated water transport. Unlike other types of diuretic agents, it selectively enhances water excretion and reduces electrolyte loss in the urine. Tolvaptan is used in Japan to control the progression of autosomal dominant polycystic kidney disease and to minimize fluid retention caused by heart failure, in situations where other diuretic agents are not successful [1–4]. This superiority in terms of clinical efficacy has prompted its use as an agent to reduce fluid retention caused by cirrhosis [5, 6]. Nevertheless, tolvaptan has several side effects, such as renal insufficiency, thromboembolism, acute hepatic insufficiency, hepatic dysfunction, shock, anaphylaxis, hepatic encephalopathy, pancytopenia, and hypernatremia. Here, we report a case of hepatocellular carcinoma (HCC) with cirrhosis, in which we administered tolvaptan after hepatectomy, ultimately leading in hypernatremia.

## Case presentation

The patient was a female in her 60s, hospitalized in the psychiatric department of a different medical institute for bipolar disorder. In April 2018, we performed a laparoscopic partial resection of segment four (S4) of the liver in our hospital, as a local treatment for HCC. Disease recurrence was identified in September 2019, during follow-up observation, and the patient was readmitted to our hospital for surgical treatment. Her medical history indicated primary biliary cirrhosis, ovarian cystoma, bipolar disorder, and cholecystolithiasis (asymptomatic). She did not have any allergies, never smoked, and only consumed alcohol occasionally. Upon admission, her body weight and body mass index were 53.0 kg and 27.85 kg/m^2^, respectively.

Contrast-enhanced computed tomography (CT) of the abdomen showed a tumor at S4 with well enhancement in arterial phase and with washout in portal phase, 27 × 18 mm in size. We also confirmed the presence of splenomegaly and splenorenal shunt but did not observe any gastric/esophageal varices or distant metastasis in the lungs or bones. In a gadolinium ethoxybenzyl diethylenetriamine pentaacetic acid-enhanced magnetic resonance imaging (MRI), we confirmed a tumor in the same region (S4), which showed high intensity in the arterial phase and low intensity in the hepatobiliary phase, adjacent to the S4 Glisson’s capsule and the hilar plate.

Preoperative blood test results revealed a low platelet count (103 × 10^3^/μL), hypoalbuminemia (3.0 g/dL), renal dysfunction (55 mL/min/1.73 m^2^ of estimated glomerular filtration rate), and an increased Na level (151 mEq/L) (Table 1).

**Table 1:** Preoperative laboratory findings

Hemoglobin	11.6 g/dL	Total bilirubin	1.1 mg/dL
White blood count	2.8 × 10^3^/μL	Na	151 mEq/L
Platelet count	103 × 10^3^/μL	Cl	118 mEq/L
Prothrombin activity	79%	K	3.9 mEq/L
Albumin	3.0 g/dL	Creatinine	0.79 mg/dl
AST	31 IU/L	eGFR	55 mL/min/1.73 m^2^
ALT	19 IU/L	C-reactive protein	0.27 mg/dL

*ALT* alanine aminotransferase, *AST* aspartate aminotransferase, *eGFR* estimated glomerular filtration rate

The indocyanine green 15 min retention test value was 49.2%, with a Child-Pugh score of 7 (class B). Preoperative chest radiography showed a 45.3% cardiothoracic ratio (CTR) with no evidence of pleural effusion.

We performed an open S4 sectionectomy in October 2019 to resect the tumor. The duration of the operation was 248 min, and the blood loss was 595 mL. The intraoperative infusion volume was 2110 mL, and we administered 480 mL of fresh frozen plasma together with 10 units of platelet concentrates. The intraoperative urine volume was 680 mL.

**Fig. 1:**
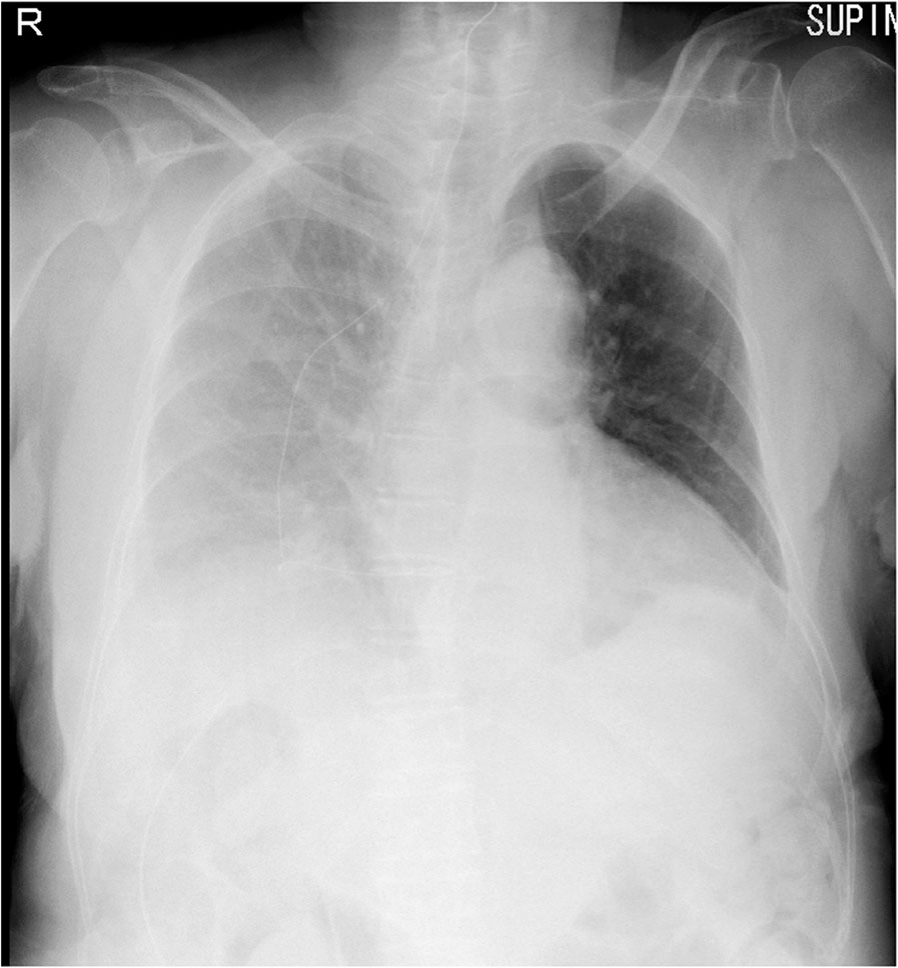
Chest X-ray findings on POD 1. We confirmed a right pleural effusion in the chest X-ray image. The CTR showed increased to 66.7%

On postoperative day (POD) 1, the patient’s level of consciousness was normal, and other vital signs were stable. Her weight increased from 53 kg (before the surgery) to 56.7 kg, and whole-body edema, as well as right pleural effusion in the chest X-ray image were evident (Fig. 1). Despite an oxygen flow of 3 L/min, her peripheral oxygen saturation (SpO_2_) level dropped to 92%. The CTR was significantly increased (66.7%) when compared to preoperative levels. We determined that this was due to the postoperative pleural effusion, and thus the patient was started on oral administration of tolvaptan and spironolactone at 7.5 and 25 mg/day, respectively. The Na level was unchanged when compared with preoperative levels (151 mEq/L).

**Fig. 2:**
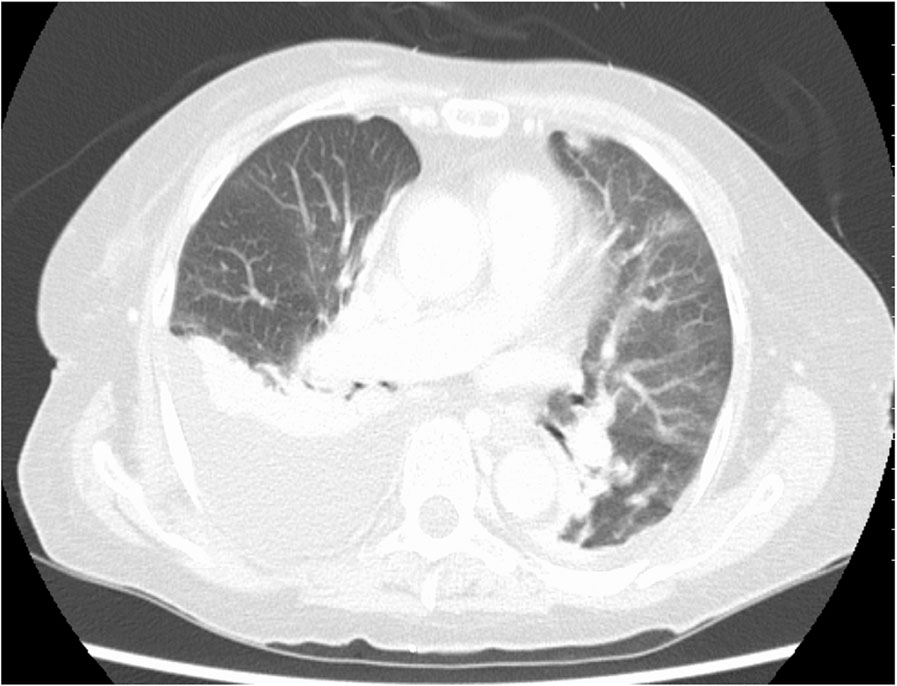
Chest CT findings on POD 2. Right pleural effusion and bilateral pneumonia were confirmed by a chest CT

On POD 2, the patient’s body temperature was 39 °C and she suffered from dyspnea and general fatigue. Since she showed delirium symptoms, anemia progression, decreased platelet count, increased inflammation, and hepatic transaminase levels, we performed a contrast-enhanced CT. Although the abdominal CT showed normal postoperative changes, the chest CT allowed us to confirm a right pleural effusion and bilateral pneumonia (Fig. 2). We then fasted the patient and started the administration of antibacterial agents (tazobactam piperacillin hydrate, 13.5 g/day). The Na level was normal on POD 2 (143 mEq/L).

**Fig. 3:**
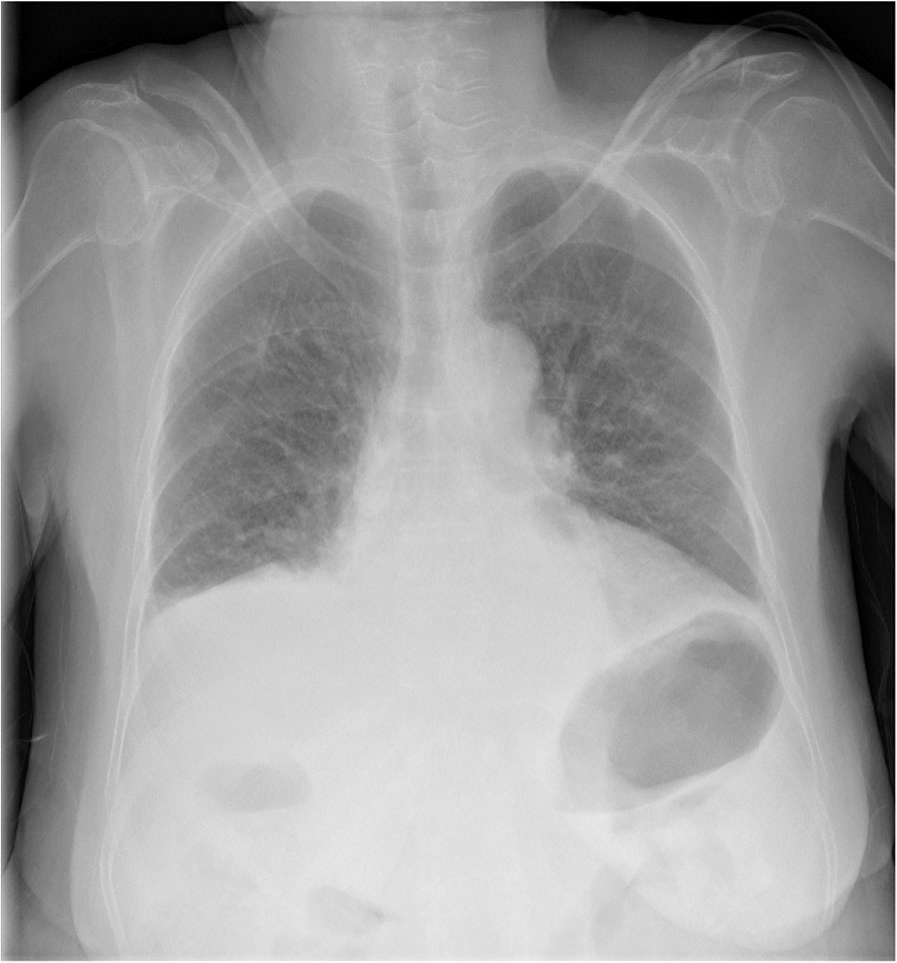
Chest X-ray findings on POD 3. The right pleural effusion improved after tolvaptan administration, and no signs of pneumonia were found

On POD 3, her body temperature subsided to 37.2 °C. She showed signs of clear consciousness, and the SpO_2_ level improved to 96% under an oxygen flow of 3 L/min. The daily urine volume was 4350 mL. The CTR did not change significantly, but there was an improvement in the right pleural effusion (Fig. 3). The Na level increased to 161 mEq/L.

On POD 4, at around 19:00, she showed disturbance of consciousness. We suspected that this condition was due to postoperative delirium, as confirmed by a psychiatrist.

**Fig. 4:**
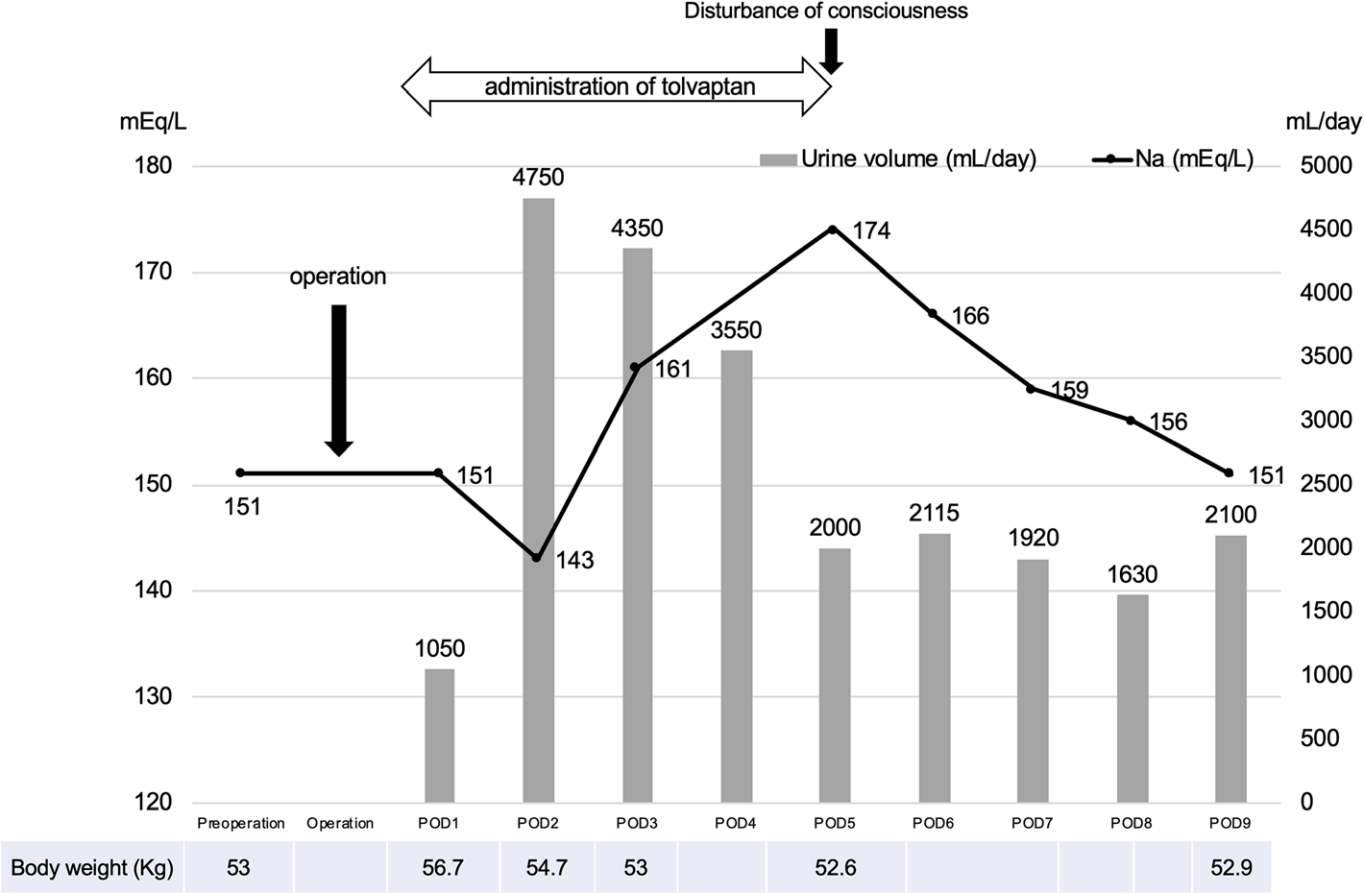
Sequential changes in serum Na level, weight, and urine volume. Tolvaptan administration was started on POD 1; the Na level, which was 151 mEq/L before the surgery, rose to 174 mEq/L on POD 5. After terminating tolvaptan administration, the Na level gradually decreased and returned to the preoperative level by POD 9. The urine volume increased after tolvaptan administration to more than 4,000 mL/day. Body weight increased by 3.7 kg compared to the preoperative level; after tolvaptan administration, it returned to values similar to those observed before the operation

The blood test on POD 5 showed an extremely high Na level (174 mEq/L), which led us to determine that the delirium was due to hypernatremia. We suspected that this could be a side effect of tolvaptan and immediately terminated its administration. The Na level gradually improved with fluid infusion therapy, dropping to preoperative levels on the ninth day; her consciousness also gradually improved (Fig. 4).

## Discussion

Tolvaptan has been used in Europe and America since the late 2000s in patients with syndrome of inappropriate secretion of antidiuretic hormone, and several studies have reported its effectiveness in heart failure, and cirrhosis with hyponatremia [7, 8]. Tolvaptan is also recommended in the treatment guidelines for cirrhosis-induced ascites or edema in Europe and America [9]. In Japan, it was only introduced in 2014 for the treatment of cirrhosis-induced ascites or edema. Several studies have reported that tolvaptan administration is safe and can reduce body weight, ascitic fluid, and edema [10, 11]. It also reduces the effective dose of loop diuretics, thereby maintaining a normal renal function [12, 13]. Tolvaptan attenuates muscle loss in patients with cirrhosis, thereby improving their prognosis and quality of life [14–16]. However, there are several side effects that should be noted, with hypernatremia being one of the most important. It occurs due to its water diuresis effect, resulting in hemoconcentration that leads to hypernatremia in 1 to 5% of cases [17, 18]. The severe cases are accompanied by disturbance of consciousness, which could lead to irreversible brain damage.

The patient developed hypernatremia following tolvaptan administration after hepatectomy for HCC. Tolvaptan was administered to deal with the exacerbation of physical status due to postoperative fluid retention. It is well known that the antidiuretic hormone vasopressin is released during the postoperative period and that those patients show symptoms of oliguria; tolvaptan has antagonist effects, which should suppress the fluid shifting to the third spaces. Therefore, tolvaptan administration was implemented to control postoperative fluid retention in hepatectomy patients with cirrhosis. The case discussed here differed from the norm, due to the relatively high preoperative Na levels, challenges in doctor-patient communication caused by the patient’s mental disorder, and the fact that the treatment was performed during a fasting period initiated due to postoperative pneumonia.

Several studies have suggested that patients, who are elderly, have renal function disorders or have elevated blood Na levels tend to develop hypernatremia after tolvaptan administration, especially at a high dosage [18–20]. A scoring system for predicting hypernatremia following tolvaptan administration has also been demonstrated (risk score = 0.125 × serum sodium (Na) + 0.032 × serum urea nitrogen (BUN)/serum creatinine (Cr) − 0.436 × serum potassium (K) + 0.014 × age) [18]. Patients with a score higher than 17.80 are considered to have a high risk of tolvaptan-induced hypernatremia. The subject of this study scored 18 and was thus in the high-risk group.

The patient had elevated preoperative blood Na level of 151 mEq/L. As mentioned above, tolvaptan may not have been appropriate in the patient because high Na levels before administration of tolvaptan have been reported as a risk factor for tolvaptan-induced hypernatremia.

The cause of preoperative hypernatremia was unknown because the patient had no complications such as diabetes insipidus and no medications that elevate Na levels before surgery. However, the patient had a history of mental illness, and less complaints of excessive thirst could be the cause of hypernatremia. The hypernatremia following tolvaptan administration was due to a combination of various factors. In particular, it may have been induced by the surgical invasion on the condition of the patient whose Na level had elevated before the operation.

The final outcome of the patient was that her Na level and consciousness improved to preoperative condition, and she was discharged from our hospital. She has been followed up periodically after discharge and has no signs of recurrence of HCC and hypernatremia.

When administering tolvaptan to high-risk patients such as this case, it is necessary to start the administration at half the recommended dose (3.75 mg/day) and check the Na level frequently.

## Conclusions

This report is the first case report of a patient who developed hypernatremia after tolvaptan administration in the early stages following hepatectomy. This demonstrates that tolvaptan administration during the early postoperative period in which the patient’s physical status is unpredictable should be carefully considered, and the patient’s status during the following period must be closely monitored.

## Abbreviations

CT: Computed tomography
CTR: Cardiothoracic ratio
HCC: Hepatocellular carcinoma
MRI: Magnetic resonance imaging
POD: Postoperative day
S4: Segment four
SpO_2_: Peripheral oxygen saturation
